# Virtual Biomarkers and Simplified Metrics in the Modeling of Breast Cancer Neoadjuvant Therapy: A Proof-of-Concept Case Study Based on Diagnostic Imaging

**DOI:** 10.3390/medsci13040242

**Published:** 2025-10-24

**Authors:** Graziella Marino, Maria Valeria De Bonis, Marisabel Mecca, Marzia Sichetti, Aldo Cammarota, Manuela Botte, Giuseppina Dinardo, Maria Imma Lancellotti, Antonio Villonio, Antonella Prudente, Alexios Thodas, Emanuela Zifarone, Francesca Sanseverino, Pasqualina Modano, Francesco Schettini, Andrea Rocca, Daniele Generali, Gianpaolo Ruocco

**Affiliations:** 1 Breast Cancer Unit, Centro di Riferimento Oncologico della Basilicata (IRCCS-CROB), 85028 Rionero in Vulture, Italy; graziella.marino@crob.it (G.M.); alexios.thodas@crob.it (A.T.); 2 Initiatives for Bio-Material Behaviour (Ibmb), 85100 Potenza, Italy; mv.debonis@gmail.com; 3 Laboratory of Preclinical and Translational Research, Centro di Riferimento Oncologico della Basilicata (IRCCS-CROB), 85028 Rionero in Vulture, Italy; marzia.sichetti@crob.it; 4 Diagnostic and Imaging Department, Centro di Riferimento Oncologico Della Basilicata (IRCCS-CROB), 85028 Rionero in Vulture, Italy; aldo.cammarota@crob.it (A.C.); manuela.botte@crob.it (M.B.); giuseppina.dinardo@crob.it (G.D.); imma.lancellotti@crob.it (M.I.L.); antonio.villonio@crob.it (A.V.); 5 Medical Oncology Unit, Centro di Riferimento Oncologico Della Basilicata (IRCCS-CROB), 85028 Rionero in Vulture, Italy; antonella.prudente@crob.it; 6 Trial Office, Centro di Riferimento Oncologico della Basilicata (IRCCS-CROB), 85028 Rionero in Vulture, Italy; emanuela.zifarone@crob.it; 7 Unit of Gynecologic Oncology, Centro di Riferimento Oncologico della Basilicata (IRCCS-CROB), 85028 Rionero in Vulture, Italy; francesca.sanseverino@crob.it; 8 Emergency and Palliative Care Unit, Centro di Riferimento Oncologico della Basilicata (IRCCS-CROB), 85028 Rionero in Vulture, Italy; pasqualina.modano@crob.it; 9 Medical Oncology Department, Hospital Clinic of Barcelona, 08036 Barcelona, Spain; schettini@clinic.cat; 10 Department of Medicine, Surgery and Health Sciences, University of Trieste, Cattinara Hospital, 34128 Trieste, Italy; andrea.rocca@units.it (A.R.); dgenerali@units.it (D.G.); 11 Multidisciplinary Unit of Breast Pathology and Translational Research, Cremona Hospital, 26100 Cremona, Italy; 12 Modeling and Prototyping Laboratory, College of Engineering, University of Basilicata, 85100 Potenza, Italy

**Keywords:** breast cancer, neoadjuvant therapy, biomarker, reactive–diffusive modeling, multidimensional modeling, diagnostic imaging, computational prognosis

## Abstract

Background: Neoadjuvant chemotherapy (NAC) is a standard preoperative intervention for early-stage breast cancer (BC). Dynamic contrast-enhanced magnetic resonance imaging (CE-MRI) has emerged as a critical tool for evaluating treatment response and pathological complete response (pCR) following NAC. Computational modeling offers a robust framework to simulate tumor growth dynamics and therapy response, leveraging patient-specific data to enhance predictive accuracy. Despite this potential, integrating imaging data with computational models for personalized treatment prediction remains underexplored. This case study presents a proof-of-concept prognostic tool that bridges oncology, radiology, and computational modeling by simulating BC behavior and predicting individualized NAC outcomes. Methods: CE-MRI scans, clinical assessments, and blood samples from three retrospective NAC patients were analyzed. Tumor growth was modeled using a system of partial differential equations (PDEs) within a reaction–diffusion mass transfer framework, incorporating patient-specific CE-MRI data. Tumor volumes measured pre- and post-treatment were compared with model predictions. A 20% error margin was applied to assess computational accuracy. Results: All cases were classified as true positive (TP), demonstrating the model’s capacity to predict tumor volume changes within the defined threshold, achieving 100% precision and sensitivity. Absolute differences between predicted and observed tumor volumes ranged from 0.07 to 0.33 cm^3^. Virtual biomarkers were employed to quantify novel metrics: the biological conversion coefficient ranged from 4 × 10^−7^ to 6 × 10^−6^ s^−1^, while the pharmacodynamic efficiency coefficient ranged from 1 × 10^−7^ to 4 × 10^−4^ s^−1^, reflecting intrinsic tumor biology and treatment effects, respectively. Conclusions: This approach demonstrates the feasibility of integrating CE-MRI and computational modeling to generate patient-specific treatment predictions. Preliminary model training on retrospective cohorts with matched BC subtypes and therapy regimens enabled accurate prediction of NAC outcomes. Future work will focus on model refinement, cohort expansion, and enhanced statistical validation to support broader clinical translation.

## 1. Introduction

Breast cancer (BC) is the most prevalent malignancy in women and a leading cause of cancer-related mortality [1]. Advances in early-stage BC management over recent decades have significantly reduced recurrence rates and improved survival [2]. However, treatment toxicity and associated costs have concurrently increased [3,4]. As recommended by the European Society for Medical Oncology (ESMO), these trends underscore the need for individualized therapeutic strategies to minimize adverse effects and optimize healthcare resource utilization [5]. The toxicity and cost associated with breast cancer (BC) treatments remain major clinical and socioeconomic challenges. Although chemotherapy and targeted regimens have demonstrated efficacy in improving disease-free survival, their administration is often accompanied by significant adverse effects that negatively affect patients’ quality of life and treatment adherence. Moreover, the high cost of prolonged and combination therapies imposes a substantial burden on both healthcare systems and patients. These limitations further underline the clinical need to develop predictive imaging and computational models capable of forecasting tumor progression and treatment responsiveness could play a crucial role in identifying patients who may safely avoid unnecessary or prolonged chemotherapy. Such approaches would support therapeutic personalization, reduce treatment-related toxicity, and contribute to more sustainable healthcare resource allocation.

Neoadjuvant chemotherapy (NAC) refers to chemotherapy administered before BC locoregional therapy with primary clinical goals of prompting tumor shrinkage, improving operability, and enabling breast conserving surgery (BCS) [6,7]. It also improves the survival of BC patients, to varying degrees depending on tumor subtype, similarly to adjuvant chemotherapy, when compared to locoregional treatment alone. NAC regimens typically combine cytotoxic agents with molecularly targeted therapies, such as anti-Human Epidermal growth factor Receptor 2 (HER2) agents or immune checkpoint inhibitors, according to BC subtype, and have demonstrated substantial clinical value in locally advanced or inoperable disease [8]. This approach enables real-time evaluation of tumor response, allowing treatment plans to be adjusted based on individual tumor behavior [9].

Personalized NAC can optimize treatment outcomes, achieving a high clinical response rate (approximately 80%) and a pathological complete response (pCR) in 6–25% of BC patients [10,11], with even higher rates (>50%) in HER2-positive and triple-negative breast cancer (TNBC) subtypes treated with anti-HER2 agents and immune checkpoint inhibitors, respectively [12,13].

Pathological response to NAC correlates strongly with disease-free and overall survival, as demonstrated by previous meta-analyses encompassing nearly 12,000 samples [12]. Furthermore, another meta-analysis demonstrated that NAC increases breast-conserving surgery rates compared to adjuvant therapy without compromising recurrence or survival [14]. Nevertheless, although disease progression is rare, approximately 20% of patients may not experience any clinical or pathological benefit from NAC [15]. Thus, accurate and timely prediction of response to NAC provide auxiliary tools in selecting the most suitable choice for each patient, among the available therapeutic options.

In addition, NAC provides a unique setting for evaluating novel therapies and investigating their effects on tumor response, improving the assessment of new drugs and treatment combinations and developing more effective treatment strategies. The evaluation of NAC effectiveness via computational models could not only drive patient treatment planning, but also predict tumor response to drugs, assist in the choice between breast-conserving surgery and mastectomy, and provide a reliable support for postoperative treatment decisions, minimizing unnecessary side effect, as suggested by Marino et al. [16]. Quantitative assessment plays a key role in evaluating BC’s response to NAC. While pathological examination remains the gold standard for evaluating tumor response, it is only performed after surgery, limiting its utility for real-time treatment personalization, predicting the pathological response, and designing a personalized NAC regimen [15]. Therefore, it is highly desirable to find a way to dynamically assess tumor response to NAC in vivo without invasive procedures so that the treatment can be adjusted at an earlier stage for both responders and non-responders.

New imaging techniques, such as dynamic contrast-enhanced magnetic resonance imaging (CE-MRI), have increasingly been employed to collect various useful information in the evaluation of NAC response, providing functional and anatomical information to predict pCR [17,18]. Meta-analyses of 5272 and 1636 patients reported pooled CE-MRI sensitivity and specificity of 0.64 (95% CI, 0.56–0.70) and 0.92 (95% CI, 0.89–0.94), respectively, demonstrating superior specificity relative to ultrasonography and mammography in predicting pCR to NAC in BC patients [15,18]. Additionally, CE-MRI, combined with positron emission tomography/computed tomography (PET/CT) or diffusion-weighted magnetic resonance imaging (DW-MRI), may further improve predictive accuracy of pCR [18].

In computational models adopting an engineering approach, tumor mass dynamics and its interaction with the drug are considered for each patient, so that valuable insights can be gained, potentially leading to improved treatment strategies and patient outcomes. Indeed, mathematical models are increasingly used in cancer research to predict how tumors develop and spread over time (spatially and temporally). Partial differential equations (PDEs) models can integrate clinical tumor growth data to simulate spatial and temporal dynamics, including diffusion and proliferation of tumors, crucial for NAC response analysis and cancer proliferation modeling. Coupled with quantitative MRI data, PDE-based models could simulate the diffusion and proliferation of various tumor components (cancer cells, healthy cells, etc.) on an individual basis [19,20,21], capturing intra-tumor heterogeneity and drug distribution within the tumor microenvironment. This in silico experimentation, featuring dosing schedules, administration routes, and drug formulations, provide a virtual laboratory for optimizing neoadjuvant therapy protocols before clinical testing, leading to more realistic predictions of therapeutic outcomes.

In a recent study, such a model was applied to a tailored simulation of an early therapeutic response to the poly ADP ribose polymerase (PARP) inhibitor olaparib in early-stage TNBC, before starting the standard NAC [22]. For each patient, clinical assessments and responses, associated with 18 F-fluorodeoxyglucose-PET/CT scans (^18^FDG-PET/CT), were conducted at baseline and after 3 weeks of olaparib. This coupled PDE framework allows examination of drug availability, penetration, and resistance mechanisms, supporting mechanistic rather than purely empirical predictions. Integration of pharmacokinetics (PK) and pharmacodynamics (PD) enhances the model’s ability to account for inter-patient variability, providing a quantitative foundation for stratified prognosis and therapy planning.

In the present study, we have applied a similar computational mass transfer approach to tumor dynamics in a retrospective selected cohort of three BC patients, who received different specific NAC regimens. By linking clinical data, clinicopathological features, personalized imaging, and mathematical modeling, this approach enables simulation of early-stage BC progression and prediction of tumor response to NAC at the individual patient level. This framework may identify novel predictive metrics to inform personalized treatment strategies and optimize therapeutic efficacy for each patient.

## 2. Materials and Methods

### 2.1. Study Design and Aim

**Figure 1:**
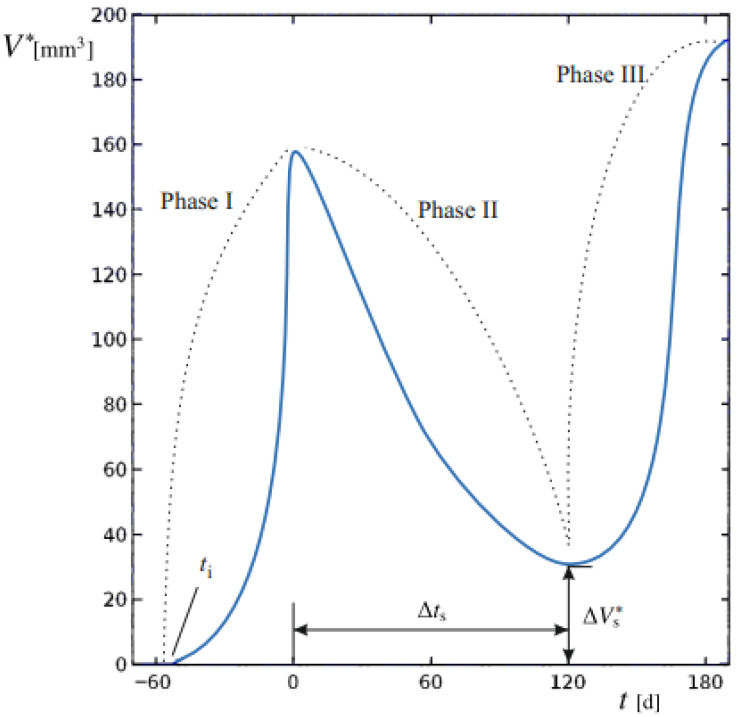
Tumor volume of a generic patient undergoing BC relapses after the end of therapy. The dynamic evolution of lesion volume V* is generally subdivided in Phases I to III, denoted by dashed lines. The starting time of the basal lesion *t_i_*, the duration of Phase II ∆*t_s_* and the residual tumor volume after NAC ∆$V_{s}^{*}$, to be removed by surgery, are also indicated.

The aim of this study was to develop and apply a mathematical modeling procedure able to reproduce the dimensional changes in primary BC in response to different NAC regimens, based on baseline and post-treatment diagnostic CE-MRI evaluations. The predicted spatial and temporal evolution of tumors evidences the different proliferation phases at the tissue scale. Computed tumor volume (V*) was compared to the clinically measured volume (*V*). The sequence of phases is reported in Figure 1 for a representative progression of V*.

We establish *t* = 0 as the time of cancer diagnosis and the initiation of the NAC regimen. During Phase I, the tumor mass, originating from an initial lesion at an unknown time t*_i_* in the past, undergoes unrestricted proliferation. At *t* = 0, baseline imaging provides a first reference for comparison with numerical model outputs. Phase II begins with NAC administration, during which tumor proliferation is challenged by the therapy regimen. At this stage, changes in V* reflect the interplay between therapy-induced regression and tumor growth dynamics. Spanning a duration *∆t_s_*, successful therapy leads to partial regression; at the end of this period, surgery would eventually be performed. A final CE-MRI performed immediately at the end of NAC and before surgery, provides the second reference point for validating the model and comparing its numerical outputs with the true tumor volumes, ensuring overall model validity.

Upon completion of therapy, a residual tumor mass of volume (∆V_s_*) may persist, potentially supporting relapses if it exceeds a critical threshold. This marks Phase III, characterized by the possibility of renewed uncontrolled growth unless surgically removed.

This work specifically aims to identify novel biomarkers for the BC disease. Clinical biomarkers (CBs) are measurable indicators derived from patient data, typically obtained through clinical assessments, imaging studies, or laboratory tests. As outlined by Perera-Bel et al. [23], these biomarkers serve multiple roles: prognostic markers, predicting disease progression; predictive markers, stratifying patient cohorts according to treatment response; and personalized markers, guiding the tailoring of therapy to individual needs.

Here, we propose to complement existing clinical metrics with virtual biomarkers (VBs), which are model-derived parameters that capture underlying biological processes or tumor characteristics. These VBs function as prognostic, predictive, and personalized markers. In particular, we aim to extract suitable VBs values for each individual patient from the computational progress analysis of Phases I and II. Conceptually, VBs act as computational counterparts to traditional biomarkers: while molecular biomarkers are experimentally measured from tissue or blood samples, VBs are inferred through mathematical modeling and describe patient-specific physiological or pharmacodynamic behavior (e.g., tumor proliferation rate or drug response). This framework enables the integration of heterogeneous clinical and imaging data into a unified, model-based representation of disease dynamics, supporting individualized prediction and therapy stratification.

### 2.2. Inclusion Study Criteria

Retrospective data from three random patients were employed in this study.

#### 2.2.1. Patients Enrolled and Therapies Administered

**Figure 2:**
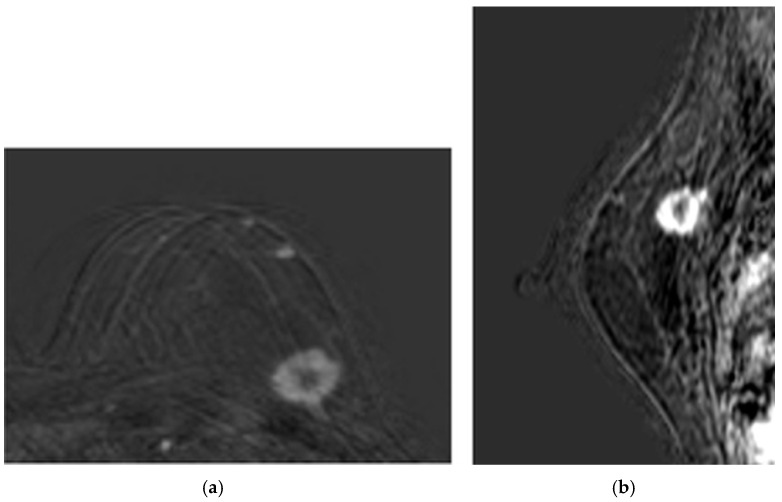
Baseline CE-MRI of Patient 1: (**a**) sample axial projection; (**b**) sample sagittal projection. The lighter areas, on the gray/black background, depict the tumoral ROIs.

**Figure 3:**
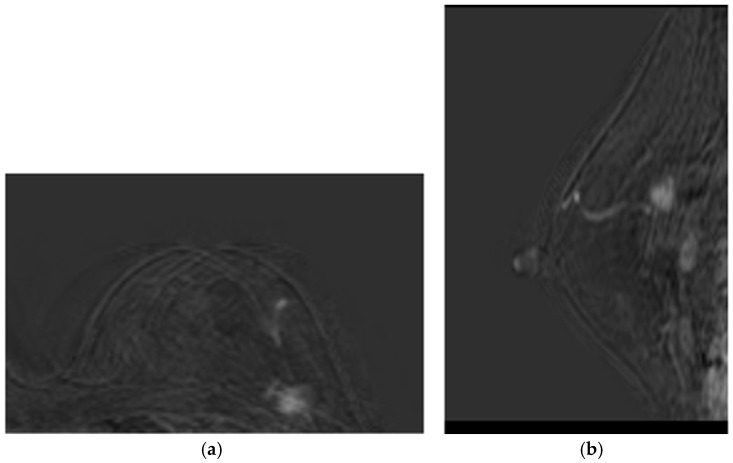
Post-NAC CE-MRI of Patient 1: (**a**) sample axial projection; (**b**) sample sagittal projection. The lighter areas, on the gray/black background, depict the tumoral ROIs.

- Patient 1 was a 60-year-old woman with both estrogen and progesterone receptors expression of 85%, nuclear protein Ki67 index of 20–25%, and HER2 amplification. Preoperative CE-MRI revealed the presence of a nodular area in the left upper outer quadrant, with a volume (*V*) of approximately 4.1 cm^3^ (Figure 2). The patient underwent NAC with epirubicin/cyclophosphamide (EC) administered intravenously (IV) every three weeks for four cycles, followed by a combination of weekly IV paclitaxel plus trastuzumab for 12 weeks. Post-NAC CE-MRI showed a reduction in the heterogeneous nodule volume (*V*) to approximately 0.18 cm^3^ (Figure 3). Clinical stage cT1 N1 M0. The patient underwent quadrantectomy with sentinel lymph node biopsy, achieving pCR (ypT0 ypN0).
- Patient 2 was a 70-year-old woman with a 3.5 cm^3^ breast tumor adjacent to the nipple-areola complex, staged cT2 N0 M0. Hormone receptor profile showed estrogen receptor 90%, progesterone 45%, Ki67 30–35%, and HER2 1+. Preoperative CE-MRI showed a heterogeneous nodule of approximately 3.5 cm^3^ with infiltrative streaks extending to the skin along the outer aspect of the gland. The patient received NAC consisting of EC × 4 (every 21 days) followed by paclitaxel × 12 (every 7 days). Post-NAC CE-MRI revealed a reduction in the volume (*V*) of the heterogeneous nodule, given by 2.5 cm in anterior–posterior dimension, 1.8 cm in diameter, and 1.2 cm in lateral dimension, compared to the initial control V measurement of 3.5 cm × 3.2 cm × 2.6 cm. The lesion currently exhibited central necrosis with fewer infiltrative streaks and no skin retraction. Following multidisciplinary discussion, the patient underwent radical mastectomy according to the Madden technique. Pathological response ypT1c(m) ypN0(i)(sn).
- Patient 3 was a 56-year-old woman with a IIIB breast tumor, staged cT4 N0 M0, both estrogen and progesterone receptors expression of 85%, Ki67 10%, and HER2 negative. Preoperative CE-MRI identified the presence of a nodular area with irregular margins (BIRADS5) in the lower inner quadrant, measuring 1.3 cm in anterior–posterior dimension, 1.4 mm in craniocaudal diameter, and 1.3 mm in lateral dimension. The lesion extended distally to the subcutaneous plane of the mammary sulcus, which appeared retracted, and was located approximately 1.6 cm from the underlying muscle plane. The patient received NAC consisting of EC × 4 (every 21 days) followed by paclitaxel × 12 (every 7 days). Post-NAC CE-MRI showed a reduction in the heterogeneous nodule in the lower-inner quadrant of the left mammary sulcus. The size remained unchanged with weaker enhancement, suggesting a partial reduction in biological vitality. The lesion had a core size of approximately 1.6 cm and presented infiltrative streaks radiating approximately 2.0 cm, reaching and retracting the skin. There were no signs of infiltration into the underlying muscle plane. Following multidisciplinary discussion, the patient underwent radical mastectomy according to the Madden technique. Pathological response ypT4b N1(sn).

#### 2.2.2. Ethics and Consent to Participate

This work was conducted in accordance with the Declaration of Helsinki [24] and the Good Clinical Practice principles [25]. The clinical study received a favorable opinion from the Regional Single Ethics Committee for Basilicata on 15 June 2021 (CEUR Approval n.1382; Prot. TS/CEUR 20210027022) and was recorded in the Institute’s General Protocol on 17 June 2021 (no. 2021-0005027). All patients provided written informed consent for participation.

### 2.3. Analysis and Transformation of Diagnostic Images

In the reported activity, the support of radiologists proved pivotal within the interdisciplinary team, allowing them to acquire and interpret diagnostic images essential for initiating the workflow sequence. The interpretation and transformation of diagnostic images serve as the cornerstone of the predictive modeling framework.

The diagnostic imaging process was performed using a Philips Achieva 3 Tesla MRI system (Philips Healthcare, Amsterdam, The Netherlands), equipped with a dedicated 16-channel coil designed for imaging patients in the prone position. This high-field-strength MRI system provides superior image quality and resolution, essential for the detailed evaluation of breast tissue and identification of abnormalities [26].

The imaging protocol included a series of specialized sequences tailored for comprehensive BC assessment:

- T2- and T1-weighted Turbo spin echo to provide high-contrast images of the breast tissue, highlighting differences in water content between various tissues [27]. It is particularly useful in differentiating between fatty and non-fatty tissues, making it easier to detect tumors and other pathologies, and for visualizing cysts, edema, and other fluid-filled structures within the breast.
- Short-TI Inversion Recovery, designed to suppress fat signals, enhancing the visibility of lesions and abnormalities within the breast tissue.
- Diffusion-Weighted Imaging with Apparent Diffusion Coefficient Maps, providing insights into the diffusion characteristics of water molecules within the tissue. Maps were generated to quantify the degree of diffusion restriction, which can help differentiate between benign and malignant lesions based on their cellular density.
- Dynamic Contrast-Enhanced implementation with enhanced T1 High-Resolution Isotropic Volume Examination Sequences, following the injection of a contrast agent, to capture the dynamic uptake and washout of contrast, allowing for the assessment of tumor vascularity and perfusion. This sequence is crucial for evaluating the angiogenic activity of tumors and identifying areas of rapid contrast uptake that may indicate malignancy.

The MRI examinations yielded results such as those shown in Figure 2 (baseline assessment) and Figure 3 (post-therapy). Following image acquisition, advanced post-processing techniques were applied to enhance diagnostic accuracy, specifically Maximum Intensity Projection (MIP) and 3D image subtraction. MIP generated 3D projections of the highest intensity values within the dataset, thereby improving visualization of contrast-enhanced structures. Simultaneously, subtraction algorithms removed pre-contrast data from post-contrast images, thereby highlighting areas of enhancement and facilitating the detection of lesions.

**Figure 4:**
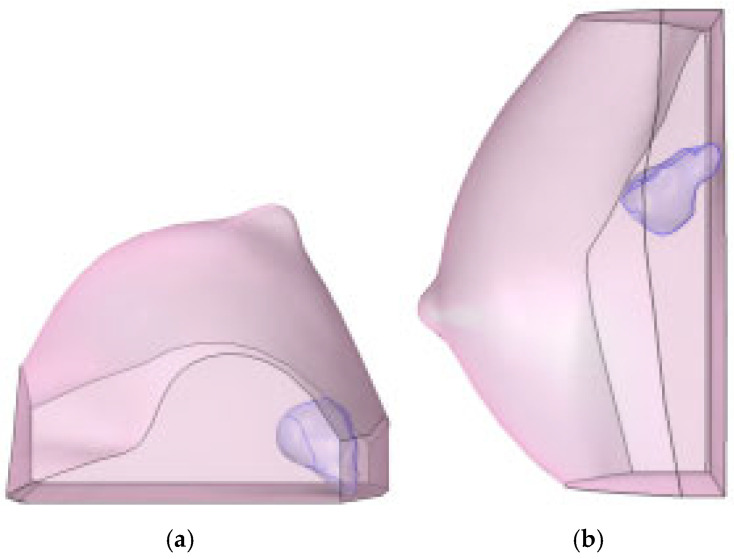
Baseline computational volumes of Patient 1’s breast, as reconstructed from the corresponding DICOM stack: (**a**) axial view; (**b**) sagittal view. The violet volumes on the pink background represent the tumoral ROIs identified in Figure 2.

**Figure 5:**
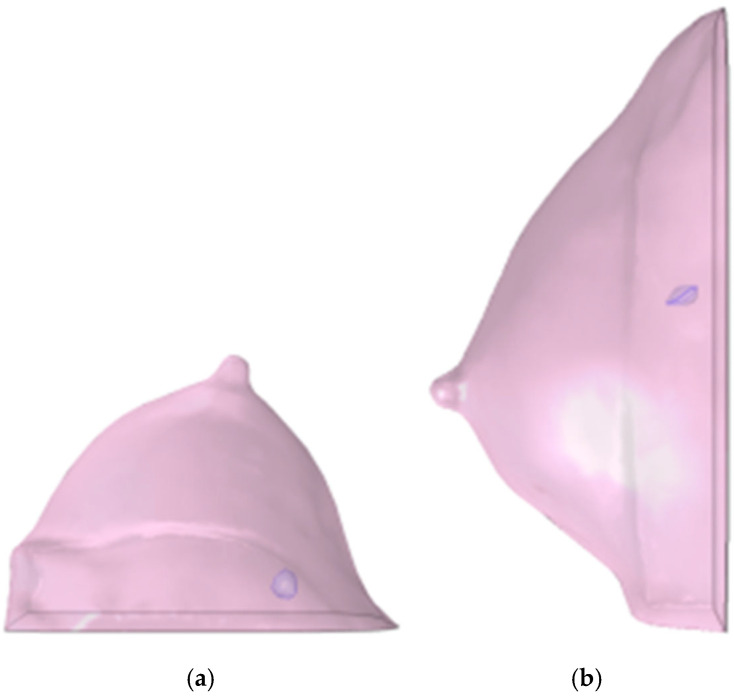
Post-NAC computational volumes of Patient 1’s breast, as reconstructed from the corresponding DICOM stack: (**a**) axial view; (**b**) sagittal view. The violet volumes on the pink background represent the tumoral ROIs identified in Figure 3.

This diagnostic imaging workflow produced DICOM (Digital Imaging and Communications in Medicine) files, which were subsequently processed for segmentation and 3D reconstruction using 3D Slicer v.4.1 [28], an open-source platform routinely employed for medical image informatics and analysis, as reported by Kikinis et al. [29]. Volumes and regions of interest (ROIs) were derived from pre- and post-contrast CE-MRI data (Figure 2 and Figure 3) by scalar volume mapping and volume rendering, as illustrated in Figure 4 and Figure 5, respectively.

**Figure 6:**
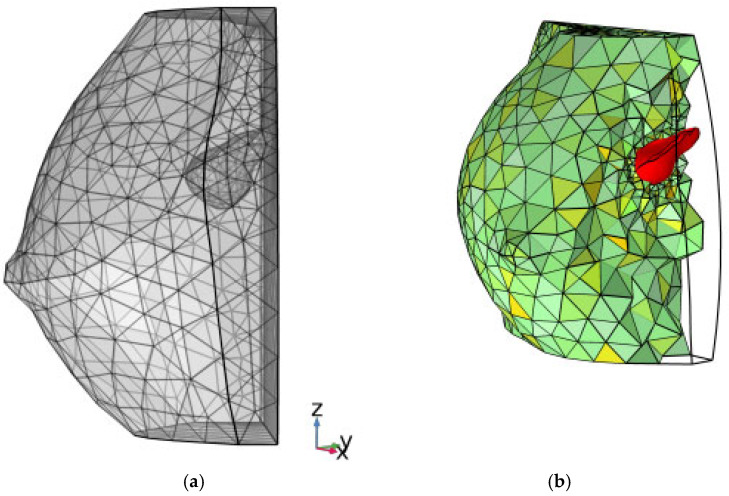
(**a**) finite element rendering of Figure 4b; (**b**) color rendering, reporting a slight rotation about the *z*-axis, where a filter is applied to computational finite elements to show the inner consistence of the grid. The baseline tumoral ROI is evidenced in color red.

The finite-element framework allowed visualization of the computational grid and the internal position of the lesion (Figure 6). Finally, 3D tumor model control volumes were exported to the computational software COMSOL Multiphysics v.5.6 [30] in STL (stereolithography) format, enabling the subsequent application of partial differential equations (PDEs) for analytical development.

### 2.4. A Diffusive-Reactive Model of Free and Challenged Tumor Growth

#### 2.4.1. Model Simplifications

As explained by Marino et al. [16], the physical and functional lag associated with multiple compartments mediating drug delivery to the tumor lesion is not considered, since the effect of mediating compartments must be approximately constant within the same patient. Given that the clinical study spans several months, the influence of such mediating compartments on macroscopic drug transfer (and the associated delay) can therefore be neglected.

Similarly, the detailed action of the extracellular matrix is not considered in this model. The crosstalk between cancer-associated fibroblasts and tumor cells has been included in the bulk effect through an effective diffusion coefficient of tumoral biomass. Likewise, the effect of microenvironmental factors, such as immune cells and fibrosis, is also omitted for simplicity. Finally, tumor heterogeneity, including variations in cell density and vascular distribution, is not explicitly accounted for.

#### 2.4.2. The Biological Conversion Mechanism

In this work, BC is a single-phase (solid) biomaterial featuring a cancer cell species that grows and invades the ROI [31,32]. Moreover, during therapy, up to *n* drug species are administered: in the BC context, *n* can be generally up to 3. When *ϕ_c_* represents the cancer cell density, or dimensionless volume in the biological matrix, Gompertzian logistics can be employed to describe the evolution (change rate) of cell density:

(1) $$\frac{d\phi_{c}}{dt}=-r_{c}\phi_{c}\ln\left(\frac{\phi_{c}}{K}\right)$$

where 1/*r_c_* is a timescale constant and K is the carrying capacity of the biological matrix, or the available ROI.

#### 2.4.3. Governing Equations

The governing PDE for reaction–diffusion of tumoral biomass transport, in space *x* and time *t*, can be applied to the ROI in terms of dimensionless cell density:

(2) $$\frac{{\partial \phi }_{c}}{\partial t}=\nabla \cdot [D_{c}(x,t)\nabla \phi_{c}]+R_{c}$$

as well as the *n* governing PDEs of drug transport, in terms of drug concentration, with *j* a drug counter:

(3) $$\frac{{\partial \phi }_{dj}}{\partial t}=\nabla \cdot [D_{dj}(x,t)\nabla \phi_{dj}]+R_{dj}$$

In Equations (2) and (3), Ds are the effective diffusion coefficients for each species/drug, while a special strategy has been devised for source terms *Rs*. Equation (3) models explicitly drug transfer in the ROI, which is missing from leading literature in computational prognosis of BC [19,20,21].

#### 2.4.4. Lesion Volume Computation, and Initial and Boundary Conditions

Due to the solution of the PDE system described by Equations (2) and (3), *ϕ_c_* and *ϕ_dj_* will be the transient distribution of dimensionless BC cell density and drugs, respectively. In particular, the lesion volume V* will inflate or deflate accordingly in ROI’s volume *Ω*_0_ and can be compared to *V* values measured by CE-MRI. From the nature of the conserved variables, V* is obtained, at any time, by integrating the *ϕ_c_* distribution in the ROI:

(4) $$V^{*}(t)=\int_{\Omega_{0}}\;\phi_{c}(t)dx$$

As *t_i_* is placed in an unknown past, it must be determined by means of minimization of the deviation of V* at *t* = 0 with respect to the preoperative *V* measurement for each given patient. Then an initial condition for *ϕ_c_* must be assumed, such that:

(5) $$V_{i}^{*}=\int_{\Omega_{0}}\;\phi_{ci}dx$$

whereas a value of *ϕ_ci_* small enough is allowed, for disease onset.

Finally, *Ω*_0_ is wrapped up in the *∂Ω_e_* surface, which can be subject to a selective permeability condition with respect to *ϕ_dj_*, so that the drug can be naturally disposed of to the ROI-confining organs; but in the present work, no *ϕ_dj_* outflow through *∂Ω_e_* is assumed.

#### 2.4.5. Numerical Treatment

The system of Equations (2) and (3) applied to *Ω*_0_, supplemented by the source terms defined by the VBs identified by running the models, along with their initial and boundary conditions, has been solved for each considered patient with the Finite Element mathematical method [30]. After a grid independency test, a final mesh of about 4k elements was employed in each case to optimize result accuracy and computational times. Execution durations, for each run, did not exceed 20 min on a Pentium Xeon server (Windows 10 Server OS, Eightcore-32N at 2.4 GHz, 128 GB RAM) in serial mode.

## 3. Results

### 3.1. The Virtual Biomarkers Defining the Source Terms

First of all, the conservative nature of Equations (2) and (3) requires that creation/destruction of *ϕ_c_* and *ϕ_dj_* must be specified, respectively:

(6) $$R_{c}=-r_{c}\phi_{c}\ln\left(\frac{\phi_{c}}{K}\right)-\;\sum_{j}^{n}\left(\epsilon_{PDj}\phi_{dj}\right)$$

(7) $$R_{dj}=f_{j}(t)m_{d}-\epsilon_{PKj}\phi_{dj}$$

**Figure 7:**
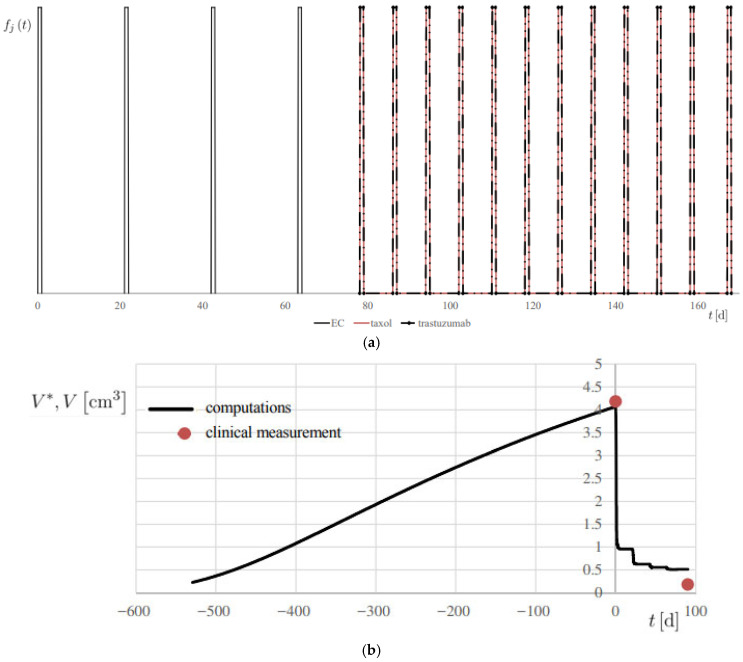
Simulation of Patient 1. (**a**) therapy modulation function *f_j_* (*t*), for each *j*-th drug; (**b**) progress of computed lesion volume V* and comparison with the associated clinical measurement *V*, using the metrics reported in Table 1.

The following definitions and assumptions apply:

- *r_c_* is a biological tumor conversion rate, a first VB of interest in this study. In this work, *r_c_* reflects the macroscopic classifications of BC for each patient, derived from the combination of histopathological type, grade, and stage of the tumor, and expression of estrogen and progestin receptors and HER2. Moreover, *r_c_* is linked to the patient’s baseline Ki67 value, integrating these clinical and molecular factors into a single quantitative descriptor of tumor aggressiveness.
- $\epsilon_{PD_{j}}$ is a mass-mediated drug efficiency, or PD behavior of the *j*-th drug, another VB of primary interest in this study. Biologically, this parameter characterizes the personalized tumor response to neoadjuvant chemotherapy (NAC). Like $r_{c}$, $\epsilon_{PD_{j}}$ reflects the patient-specific biological sensitivity to treatment, as it quantifies the temporal reduction in tumor volume induced by each administered drug. This parameter is particularly relevant for stratifying patients and identifying the most effective therapeutic regimen.
- *f_j_ (t)* is the personalized regimen indicator, or therapy regimen function, for each *j*-th drug, based on the specification outlined in Section 2.2.1, and reported, for example, in Figure 7a, for patient 1.
- *m_d_* is the actual administered mass flow rate of the therapy drug, determined on a personalized basis.
- $\epsilon_{PK_{j}}$ is the known effect of the clearance, or PK behavior, for each *j*-th drug, generally depending on each patient’s weight and body surface area and the available drug specs.
- The effective diffusivities *D_c_* and *D_dj_* had the same values, in this proof-of-concept study, for all considered patients.

To perform a proof-of-concept of the numerical procedure, we identified a useful set of VBs as listed in Table 1, with no sources of error affecting their validity and generalizability. For $\epsilon_{PDj}$, an aggregated value $\epsilon_{PD}$ was derived. It is seen that both *r_c_* and $\epsilon_{PD}$ VBs were found to lie within well-defined ranges: 2 orders of magnitude for *r_c_* and 3 orders of magnitude for $\epsilon_{PD}$.

The two virtual biomarkers selected in this study—$r_{c}$ and $\epsilon_{PD}$—were chosen because they capture two fundamental and complementary aspects of tumor dynamics: the intrinsic proliferative potential of the cancer tissue and its pharmacodynamic response to therapy. Together, these parameters provide a mechanistic and clinically interpretable representation of tumor behavior under treatment.

The optimal values for such VBs were determined through a twofold approach:

1. Individualized optimization: For each patient, the model was calibrated from the available CBs and imaging data to minimize the discrepancy between the quantitative predicted tumor volume obtained through the computational framework and the CE-MRI-derived measurements of the cancer lesion at baseline and post NAC. This process involves model training, i.e., adjusting the corresponding VB value to match observed CB, thereby improving the accuracy of the computational model in predicting tumor behavior at the individual level.
2. Cohort-based consistency: To ensure reproducibility and extract clinically meaningful patterns, optimized VB values were then analyzed across patient subgroups sharing clinical or molecular characteristics. This step enabled the identification of clinically relevant insights consistent within each subgroup, enhancing the robustness of the model and its potential for stratifying patients in future applications.

### 3.2. Simulations of the Clinical Volume

Model training was performed by integrating Equations (2) and (3) for each patient. Initially, trial values for *r_c_* and *t_i_* were tested to achieve superimposition of computed volume V* with the clinical volume *V* at *t* = 0. A second fit at *t* = *∆t_s_* was obtained by keeping the identified *r_c_* value while testing different validating values for $\epsilon_{PD}$. Modeling from the beginning of Phase I was essential to establish the cell budget within the ROI, which was subsequently validated at *t* = 0.

**Figure 8:**
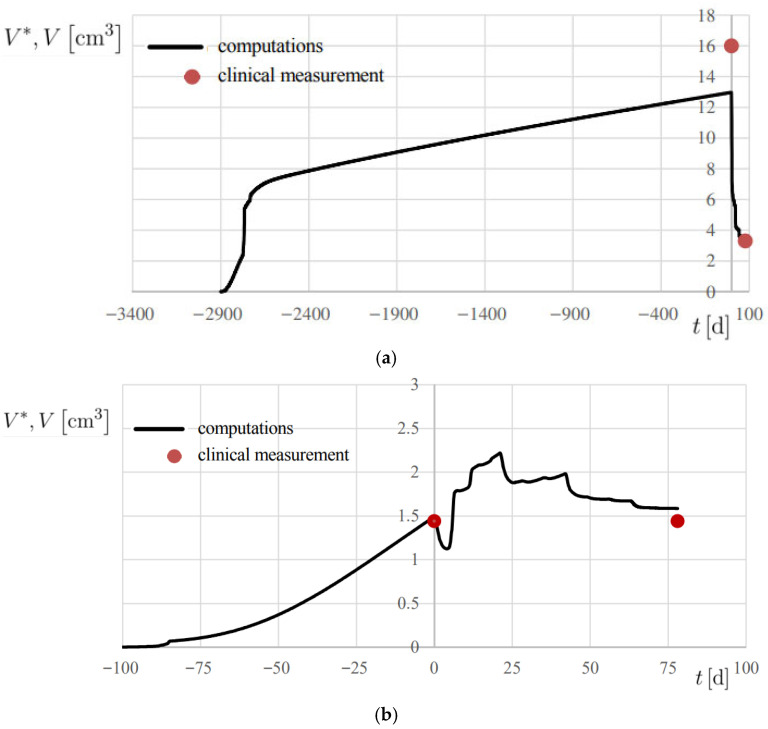
Progress of computed lesion volume V* and comparison with the associated clinical measurement *V*, using the metrics reported in Table 1. (**a**) Patient 2; (**b**) Patient 3.

The results are reported in Figure 7b and Figure 8, showing the temporal evolution of V*, and numerical results are also summarized in Table 2. A 20% error margin was applied to assess computational accuracy, with validation based on absolute differences between predicted and observed volumes. Overall, predictions were highly accurate, except at *t* = 0 for Patient 2 (Figure 8a), where an absolute difference of 3.02 cm^3^ was observed.

All cases were classified as true positive (TP), demonstrating the model’s ability to correctly predict tumor volume changes within the defined threshold. Accordingly, precision and sensitivity were both evaluated 100%, confirming model reliability. Specificity, however, could not be assessed due to the lack of negative cases in the current cohort; further validation in a more heterogeneous dataset, including poor responders, will be necessary to assess this classification metric.

In all patients, the model successfully reproduced both the initial onset of the disease in its free proliferation dynamics of Phase I and the therapy-challenged proliferation of Phase II, where treatment effects were clearly evident (Figure 1).

## 4. Discussion

The models developed in this study were designed to achieve the highest possible accuracy, measured as the difference between baseline and post-NAC lesion dimensions obtained from CE-MRI and the associated simulated values at the same corresponding times. Training the model on retrospective patient data enabled us to define value ranges for two principal virtual biomarkers (VBs), demonstrating the model’s flexibility. This framework can be extended to future cohorts sharing the same BC subtype and therapy, while also accommodating heterogeneity in tumor molecular profiles and treatment pharmacodynamics.

NAC plays a central role in reshaping surgical strategies for BC patients, particularly by increasing eligibility for breast-conserving surgery (BCS) and improving patient outcomes [33,34,35]. However, accurately predicting response to NAC and long-term treatment success remains a significant challenge. The novelty of this work lies in the integration of patient-specific clinical data, CE-MRI imaging, and reaction–diffusion computational modeling to derive biologically interpretable virtual biomarkers ($r_{c}$ and $\epsilon_{PD}$), providing a mechanistic, patient-tailored prediction of tumor response. Computational models such as the one presented here—by integrating patient-specific clinical data, mathematical models, and advanced imaging techniques—offer the potential for dynamic, personalized prediction of tumor response, thereby supporting the refinement of treatment strategies.

Validated computational models could also enhance monitoring and follow-up strategies for BC patients [36,37]. By simulating tumor evolution and therapeutic response, such models may help predict long-term outcomes and recurrence risk, guiding the frequency and intensity of post-treatment surveillance. This, in turn, would facilitate earlier detection of relapse or metastasis and enable timely interventions to improve survival [36].

With improved monitoring, clinicians can initiate appropriate interventions, leading to better patient management and improved survival rates. In addition, these models could inform shared decision-making between clinicians and patients. By providing personalized predictions of treatment response, they can enable informed discussions on therapeutic options, risks, and expected outcomes. Such patient engagement may improve satisfaction, adherence, and ultimately treatment efficacy [38,39,40].

The computational model described in this study simulates tumor growth and response to NAC, with the potential to predict treatment outcomes. A critical aspect of such modeling is the selection of parameter values, which depend on both the biological characteristics of the neoplasm and the pharmacodynamic (PD) and pharmacokinetic (PK) properties of the administered drugs [41]. Particularly important in this model are the tumor conversion rate (*r_c_*) and the PD efficiency ($\epsilon_{PD}$).

*r_c_* is a tumor growth-related parameter that reflects the complex of histopathological and biomolecular features, such as histological type, grade, hormone receptor expression, HER2 status, and proliferative index, that collectively determine the aggressiveness of the tumor. In contrast, $\epsilon_{PD}$ reflects the therapeutic activity of each anticancer drug, or drug combination, on a specific tumor subtype. In this proof-of-concept application, both parameters were estimated empirically to calibrate the model and optimize accuracy. Importantly, their values varied within a limited range across patients, which is plausible given that all tumors were hormone receptor–positive and treated with the same chemotherapy regimen, although one patient had HER2-amplified BC.

The development of this type of model serves multiple purposes: accurately predicting response to NAC, guiding the selection of the most effective regimen for each patient, and optimizing the timing and type of locoregional treatment. Achieving these goals requires identification of parameter values specific to tumor subtypes and to the PK and PD characteristics of the drugs or drug combinations employed [41]. Equally important is validation of model accuracy in larger retrospective cohorts—including different BC subtypes and treatment regimens—as well as in prospective studies. By evaluating tumor dynamics before and after NAC, the model predicts the likelihood of achieving a pathological complete response (pCR) or if residual disease will persist. This insight is crucial, as pCR is strongly associated with favorable outcomes and eligibility for BCS, whereas non-responders may require more extensive surgery, such as a mastectomy [42]. The identification and use of virtual biomarkers in this context represent a novel approach, linking computational predictions to clinically measurable tumor characteristics and therapeutic response.

Previous studies [19,22] further highlight the utility of such models, demonstrating their ability to simulate tumor responses to anticancer agents and assist in surgical planning by quantifying tumor shrinkage. For example, the residual tumor volume (∆V_s_*) at the end of Phase II (*t* = *∆t_s_*) could provide an objective basis for determining the optimal timing of surgery. In addition to improving the accuracy of surgical planning, these models may refine the extent of tissue resection in BCS. For instance, if NAC induces significant tumor shrinkage, the computational model can predict the minimal amount of tissue that needs to be removed to ensure clean margins, thereby preserving breast tissue and improving cosmetic outcomes. Conversely, if the model predicts that NAC will not sufficiently reduce the tumor size or achieve clear margins, the surgeon may opt for mastectomy upfront, reducing the risk of incomplete resection or secondary surgeries [35].

Incorporating advanced imaging modalities such as CE-MRI and PET/CT into computational models further enhances predictive precision. Previous studies have demonstrated that these techniques provide superior sensitivity and specificity in predicting pCR compared with conventional methods [15,18,42]. When integrated into modeling frameworks, they enable real-time monitoring of tumor response, allowing clinicians to dynamically adjust surgical strategies during NAC. Moreover, these models provide a platform for evaluating the effectiveness of novel drug regimens and combination therapies in virtual clinical trials using data from real-world clinical settings. By simulating multiple treatment scenarios and their effects on tumor dynamics, the proposed proof-of-concept model supports the identification of the most effective chemotherapy regimens tailored to individual patients. The integration of imaging-derived data with mechanistic modeling represents a novel contribution of this work, enabling patient-specific simulations and prediction of treatment outcomes in silico.

Despite these promising insights, several limitations must be acknowledged, that may impact on the generalizability and reproducibility of the results. This retrospective analysis was performed on a small dataset comprising only three cases. While these cases provided valuable information for preliminary model development and validation, the limited sample size restricts the model’s statistical power and its potential to generalize to broader patient populations. Nonetheless, the retrospective design allowed direct comparison of model predictions with actual tumor response observed in clinical data, providing a valuable proof-of-concept validation.

Application of the model to larger and more diverse cohorts will be essential to robustly assess predictive accuracy and clinical utility. Expanding the dataset will also allow for more comprehensive evaluation of variability within and across patients. In addition, the ad hoc estimation of VB values may not fully capture tumor heterogeneity. Future research should refine parameter estimation through systematic and statistically rigorous approaches, ideally informed by larger datasets, to improve accuracy and reproducibility.

Integrating computational models with imaging data holds strong potential to define individualized treatment strategies upfront for patients with early or locally advanced BC. With further validation and refinement, such models may become indispensable tools in modern BC management, enhancing both oncological safety and patient quality of life.

## 5. Limitation and Future Perspectives

Despite the promising proof-of-concept results, several limitations of this study should be acknowledged. First, the analysis was conducted on a small retrospective cohort of only three patients, all presenting the same breast cancer subtype (ER+, PR+). While this homogeneity was essential to ensure model stability and evaluate feasibility, it limits the generalizability of our findings to other subtypes, molecular profiles, and treatment regimens. Second, the current framework may not fully capture tumor heterogeneity or the complexity of interactions within the human organism, including microenvironmental influences such as extracellular matrix composition, vascular distribution, and stromal cell interactions. Third, the predictive performance of the simulations inherently depends on the quality and completeness of input data; missing, biased, or unrepresentative data could compromise model reliability. Finally, the retrospective nature of data collection resulted in variable timing between imaging and treatment steps, preventing the establishment of an optimal temporal window for prediction. Nevertheless, an advantage of the retrospective design is that it allows direct comparison between the predictions of our computational model and the tumor response observed in clinical data. This provides a valuable opportunity to evaluate the internal consistency and feasibility of the proposed framework, even within a small, proof-of-concept cohort, before extending the approach to prospective studies.

Future research will focus on expanding the patient cohort to include a larger and more diverse population, encompassing multiple breast cancer subtypes, molecular profiles, and therapeutic regimens, to allow for rigorous statistical validation and generalization. Parameter estimation will be refined using data-driven approaches, and the framework will be extended to integrate multimodal imaging, molecular, and histopathological data. Additionally, microenvironmental factors and tumor heterogeneity will be explicitly incorporated to improve the biological fidelity of predictions. Prospective studies with standardized imaging intervals and longitudinal follow-up will support clinical translation, aiming to establish VBs as reliable decision-support tools for personalized treatment planning and therapy optimization. Furthermore, simulating virtual clinical trials could provide a valuable platform for evaluating novel therapeutic strategies and optimizing individualized treatment regimens in silico before clinical implementation.

## 6. Conclusions

This proof-of-concept study demonstrates the feasibility and potential clinical value of integrating contrast-enhanced MRI (CE-MRI) with reaction–diffusion computational modeling to predict breast cancer response to neoadjuvant chemotherapy (NAC). By deriving and validating patient-specific virtual biomarkers (VBs), the proposed framework achieved accurate predictions of tumor volume dynamics across retrospective cases, providing mechanistic insights into tumor growth and therapeutic response.

The identification of VBs—such as tumor conversion rate ($r_{c}$) and drug efficiency ($\epsilon_{PD}$)—highlights the translational potential of computational modeling as a prognostic, predictive, and decision-support tool in breast cancer management. Unlike previous approaches limited to single-case analyses or lacking mechanistic interpretability, this integrative model offers a quantitative, biologically grounded description of tumor dynamics that bridges clinical imaging, computational simulation, and individualized prognosis.

Although the current study is limited by its small, homogeneous cohort and empirical parameter estimation, it establishes a methodological foundation for model-assisted clinical decision-making in personalized oncology. Future work will involve expanding the cohort, refining parameter inference through data-driven and machine learning techniques, and integrating multimodal imaging and molecular profiling to enhance predictive accuracy and generalizability.

Ultimately, this novel and promising framework lays the groundwork for computationally guided oncology—where predictive modeling, grounded in patient-specific imaging and clinical data, informs neoadjuvant treatment planning, supports real-time response monitoring, and enables virtual clinical trials for emerging therapies. The integration of mechanistic modeling into clinical workflows holds promise to improve treatment personalization, optimize surgical decision-making, and enhance patient outcomes and quality of life in breast cancer care.

## Figures and Tables

**Table 1:** Values for personalized driving parameters in Equations (2)–(7).

Patient	*D_c_* [m^2^/s]	*D_dj_* [m^2^/s]	*r_c_* [1/s]	$\epsilon_{PD}$ [1/s]	$\epsilon_{PK_{1}}$ [1/h]	$\epsilon_{PK_{2}}$ [1/h]	$\epsilon_{PK_{3}}$ [1/h]
1	1.0 × 10^−13^	1.0 × 10^−5^	4.0 × 10^−7^	3.64 × 10^−6^	2	50	50
2	1.0 × 10^−13^	1.0 × 10^−5^	5.7 × 10^−6^	4.54 × 10^−4^	2	50	-
3	1.0 × 10^−13^	1.0 × 10^−5^	6.0 × 10^−6^	1.14 × 10^−6^	2	50	-

**Table 2:** Volume measurements and results. Absolute differences in AD are also reported.

Patient	*V* (*t* = 0) [cm^3^]	V* (*t* = 0) [cm^3^]	AD (*t* = 0) [cm^3^]	$∆V_{s}$ (*t* = *∆t_s_*) [cm^3^]	$∆V_{s}^{*}$ (*t* = *∆t_s_*) [cm^3^]	AD (*t* = *∆t_s_*) [cm^3^]
1	4.2	4.07	−0.13	0.18	0.51	+0.33
2	16.0	12.98	−3.02	3.3	3.37	+0.07
3	1.4	1.49	+0.09	1.4	1.58	+0.18

## Data Availability

The original contributions presented in this study are included in the article. Further inquiries can be directed to the corresponding author.

## Abbreviations

The following abbreviations are used in this manuscript:

BC	breast cancer
BCS	breast conserving surgery
CBs	clinical biomarkers
CE-MRI	contrast-enhanced magnetic resonance imaging
CI	confidence interval
DICOM	digital imaging and communications in medicine
DW-MRI	diffusion-weighted magnetic resonance imaging
EC	epirubicin/cyclophosphamide
ESMO	European society for medical oncology
HER2	human epidermal growth factor receptor 2
IV	intravenously
MIP	maximum intensity projection
MRI	magnetic resonance imaging
NAC	neoadjuvant chemotherapy
PARP	poly ADP ribose polymerase
pCR	pathological complete response
PD	pharmacodynamics
PDE	partial differential equation
PET/CT	positron emission tomography/computed tomography
PK	pharmacokinetics
ROI	regions of interest
STL	stereolithography
TNBC	triple-negative breast cancer
TP	true positive
VBs	virtual biomarkers
^18^FDG	^18^F-fluorodeoxyglucose
**Nomenclature**	
The following nomenclatures are used in this manuscript:	
*AD*	absolute volume difference, cm^3^
*D*	diffusion coefficient, m^2^/s
*f*	temporal administration pattern of therapy, or modulation function, 1/d
*K*	carrying capacity of the biological matrix, dimensionless
*m_d_*	administered mass flow rate, determined on personalized basis, mol/m^3^ s
*r_c_*	biological conversion rate, 1/s
*R*	source term in Equations (2) and (3), 1/s
*t*	time, d
*V**	computed lesion volume, cm^3^
*V*	measured lesion volume, cm^3^
*x*	coordinate vector, cm
**Greek**	
*∆t*	duration, d
*∆V*	residual lesion volume to remove after NAC, cm^3^
*ϵ_PD_*	drug efficiency (effect of drug on body), 1/s
*ϵ_PK_*	drug clearance (effect of body on drug), 1/s
*∂Ω_e_*	surface, m^2^
*ϕ_c_*	normalized cancer cell density or volume, dimensionless
*ϕ_d_*	normalized drug concentration, dimensionless
*Ω*	computational volume, cm^3^
**Subscripts**	
*0*	nominal, ROI
*c*	cancer
*d*	drug
*e*	interface with the environment
*i*	initial, starting time
*j*	drug counter
*s*	surgical
